# Superficial Zoonotic Mycoses in Humans Associated with Cattle

**DOI:** 10.3390/pathogens13100848

**Published:** 2024-09-29

**Authors:** Marcin Piorunek, Honorata Kubisiak-Rzepczyk, Tomasz Trafas, Tomasz Piorunek

**Affiliations:** 1 Veterinary Practice Marcin Piorunek, 60-185 Skórzewo, Poland; 2 Department of Dermatology, Poznan University of Medical Sciences, 60-355 Poznan, Poland; rzepczykh@ump.edu.pl; 3 Department of Medicine and Health Sciences Calisia University, 62-800 Kalisz, Poland; 4 Department of Pulmonology, Allergology and Pulmonary Oncology, Poznan University of Medical Sciences, 60-569 Poznan, Poland; ttrafas@ump.edu.pl (T.T.); tpiorun@ump.edu.pl (T.P.)

**Keywords:** cattle, zoonoses, prevalence, clinical manifestations, diagnosis, treatment, prevention

## Abstract

Dermatophytosis in cattle is most often caused by infection with *Trichophyton verrucosum* (*T. verrucosum*), but also with *Trichophyton rubrum* (*T. rubrum*), *Trichophyton mentagrophytes* (*T. mentagrophytes*) and others, regardless of the geographical zone. The infection is transmitted through direct contact between animals as well as infected environmental elements. The human-to-human transmission of fungal infection is also possible.. This retrospective study was conducted based on a detailed analysis of the results of the mycological examination and medical documentation of 40 patients from Greater Poland, diagnosed with cattle-to-human dermatophytoses from March 2017 to November 2023. *T. verrucosum* accounted for 97.5% of infections and *T. mentagrophytes* for 2.5%; no other species of dermatophytes from cattle were found. Superficial skin mycosis in humans associated with cattle was more often diagnosed in small children and men directly engaged in cattle breeding. The dominant etiological factor of the superficial fungal skin infection was *T. verrucosum*, which mainly affected the scalp in children and upper limbs in adult men. In relation to the cattle population in Greater Poland, the number of cases of superficial skin mycoses among cattle breeders and their family members over the period of more than six and a half years of observation does not seem to be high.

## 1. Introduction

Cattle population worldwide amounted to 940.37 million heads in 2022, and it has been on an increasing trend since 2019 [1]. The list of letter codes for cattle breeds includes 64 breeding breeds [2]. The number of cattle has fluctuated over the reported period between 1950 and 2022 in Poland, showing an increasing trend in beef cattle and downward trend in dairy cattle since 2016 [3]. In December 2023, the cattle population in Poland was 6,435.5 thousand, including 2,203.9 thousand cows. According to the Polish Central Statistical Office, in December 2023, the cattle population in Greater Poland amounted to almost 1,152 thousand units [4]. The vast majority of cattle breeding farms in Greater Poland are medium-sized and large family farms.

Dermatophytes causing diseases in animals and posing a threat to humans belong to the genera *Epidermophyton*, *Microsporum*, *Nannizzia*, *Trichophyton* and almost 40 other species [5]. Dermatophytosis in cattle is most often caused by infection with *T. verrucosum*, but also with *T. rubrum*, *T. mentagrophytes* and *Trichophyton simii* (*T. simii*) or *Nannizzia gypsea* (*N. gypsea*) regardless of geographical zone [6,7]. *T. verrucosum* is less common in horses and small ruminants such as sheep and goats, as well as in llamas and camels [7,8,9,10]. 

Calves aged 1–6 months, regardless of gender and breed, get sick more often than adults because they have not yet developed natural specific immunity to fungi. However, they have high skin PH [6,11,12,13]. The infection is transmitted through direct contact between animals and through infected environmental elements such as brushes, gates, feed carts, farm tools and employees, and others or through asymptomatic carriers. Inappropriate sanitary conditions, high animal concentration, frequent animal regrouping, high humidity and the temperature of cowsheds, and also, malnutrition, changes in diet, immune and vitamin deficiencies or parasitic infections and intensive use of corticosteroids contribute to the spread of the infection [13,14]. An additional factor that allows and favors the spore formation and transmission of infection is the hot and humid climate in some countries. In countries with temperate climate, the peak of infection usually occurs in summer and winter [12,15]. A connection of an increased number of fungal skin infections with a humid oceanic climate, rainy years, and an autumn and winter period, when animals are confined indoors, has also been observed [16,17]. Spores can persist in the environment for up to several years and be a potential source of infection [18]. Therefore, knowledge of the local epidemiology of dermatophytes is important for the mycologist when identifying fungi and for the physician [16]. Superficial zoonotic mycosis in humans results from direct contact with infected cattle or elements of the animal environment, and can also be transferred from human to human [13,19,20]. Clinical manifestations, methods of diagnosis, treatment, and prevention of dermatophyte infections in cattle and humans are included in Table 1.

**Table 1:** Clinical manifestation, diagnosis, treatment, and prevention of dermatophytosis in cattle and humans.

	Cattle	Humans
Clinical Manifestations	− Dermatophytosis occurs in the form of round, limited areas of baldness ranging in size from several to a dozen or so centimeters or changed, large surfaces of skin with an irregular surface, grayish-whitish in color, covered with hard and firmly adherent scabs. − Skin lesions are located on the head, neck, face and around the eyes, as well as on the sides of the trunk and back. − The habit of licking skin lesions on the face and around the eyes may be an additional source of infection. − The frequent occurrence of fungal lesions on the sides and back of cattle is explained by the greater exposure to pollution, injuries, and moisture of these body parts [12,21] (Figure 1).	− Skin changes are in the form of oval lesions on both hairy and hairless parts of the body. − Ringworm of the scalp is caused by zoophilic fungi *T. verrucosum* and *T. mentagrophytes*. − It usually presents as a painful inflammatory tumor or infiltration with numerous pustules and abundant purulent content that dries into crusts. − Within the infiltration, the hair is loosely embedded in the hair follicles and is easy to remove. When pressure is applied, purulent contents leak from the hair follicles: the “sieve sign”. − The nuchal and retroauricular lymph nodes may be enlarged (Figure 2). − The forms of mycosis ○ The mild form of mycosis vulgaris forms erythematous foci that grow centrifugally, disappear in the center of the lesion and become mildly scaly (Figure 3). ○ The disseminated form, with a greater inflammatory reaction, forms oval erythematous and exfoliative foci with numerous papules or pustules on the periphery.
Diagnosis	− Conventional diagnostic methods include direct microscopic examination using light and fluorescence microscopy and culture. − Molecular techniques, including real-time PCR and MALDI-TOF MS, are also used in the detection and identification of dermatophytes [22,23]. − The diagnostic material submitted for mycological tests consists of scrapings from the periphery of fresh skin lesions. − Microscopic examination of hair and scrapings for the presence of arthrospores involves digesting the sample with a 10% aqueous solution of KOH or NaOH for 10 min. − Culture enabling species identification of the fungus is performed on Sabouraud medium or on specialized media intended for dermatophytes at the temperature of 37 °C [24,25].	− Detailed interview (contact with cattle) − Diagnostic methods for dermatophytosis ○ Direct microscopic examination ○ Culture on Sabouraud Dextrose Agar (SDA) ○ Molecular methods
Treatment	− Treatment depends on the severity of skin lesions and the number of infected animals. − Treatment costs should be taken into account when making therapeutic decisions. − Self-healing of mycosis is possible in about four months. − In less severe cases, an ointment containing enilconazole can be applied topically. − Iodine and sulfur preparations are characterized by low effectiveness. − In some cases, to facilitate penetration of the topically applied preparation, the affected fur should be trimmed and scabs should be removed. − Systemic treatment ○ Includes antifungal drugs such as miconazole, fluconazole, itraconazole and terbinafine, which have proven effective in the treatment of skin lesions. ○ Is associated with high costs and long treatment periods, as well as toxic effects for cattle and humans as consumers [24,26]. − An alternative therapy in selected cases may be the use of natural plant extracts with antifungal properties, such as garlic, lemongrass, datura, acacia, triplex, ginger, black cumin, neem, eucalyptus, alfalfa and basil [27]. − Supplementation of vitamins A, D and zinc plays a supporting role in the treatment of skin mycosis [28,29,30,31].	− Local treatment ○ Local treatment of mycosis of the glabrous skin involves the use of creams or ointments containing antifungal drugs, such as azole derivatives (clotrimazole, miconazole, isoconazole, econazole, bifonazole, flutrimazole), allylamine (terbinafine, naftifine) and ciclopirox. ○ Treatment duration is usually 2–3 weeks. It is advisable to continue therapy for 1–2 weeks after the symptoms disappear. ○ Local treatment is supportive and includes the use of antifungal drugs in the form of solutions, gels or shampoos [32,33]. − Systemic treatment ○ In systemic treatment, fluconazole, itraconazole and terbinafine are used. ○ Treatment lasts from 2 to 4 weeks. − Causal treatment ○ In the causal treatment of mycosis of the scalp, griseofulvin and terbinafine, which are particularly effective against infections caused by fungi of the Trichophyton genus, as well as itraconazole and fluconazole, are used. ○ The treatment duration depends on the type of preparation used and is usually from 2 to 8 weeks.
Prevention	− The prevention of fungal infections and counteracting their spread include periodic epidemiological examinations, compliance with the hygiene rules, systematic cleaning and disinfection of herds and vaccination of young calves with a live strain of *T. verrucosum*, as well as the treatment of infected animals and people involved in cattle breeding [13,34,35]. − Animal vaccinations are of fundamental therapeutic and preventive importance [36,37]. − It is advisable to improve the conditions of animal breeding, including increasing access to sunlight, improving the ventilation of cattle barns (reducing humidity and ammonia concentration), using green fodder and isolating infected animals [24].	− Avoiding contact with dermatophyte-infected animals. − Using disposable gloves when handling cattle. − Frequent hand washing and disinfection. − Disinfection of equipment used for handling cattle. − Isolation and proper treatment of infected humans.

The aim of the study was to identify superficial zoonotic mycosis infections in humans directly or indirectly associated with cattle breeding, to determine dermatophyte species derived from cattle and the location of the infection in relation to the age of the patients.

## 2. Materials and Methods

This retrospective study was conducted based on a detailed analysis of the results of the mycological examination and medical documentation of 40 patients from Greater Poland, diagnosed with cattle-to-human dermatophytosis in the Medical Mycology Laboratory of the Department of Dermatology and Venereology at the Poznan University of Medical Sciences (PUMS) from March 2017 to November 2023. The mycological diagnostics consisted of detecting fragments of fungal structures directly from the material collected from the patient and fully identifying the etiological factor. The detailed procedures followed depended on the location of the skin lesions, the type of material examined, and the suspected etiological factor. 

The diagnostic material consisted of the following:

− Epidermal scales scraped off with a surgical curette or scalpel from the periphery of the fungal lesions;
− Covers of blisters or vesicles and swabs from under the cover and deep swabs from the diseased places;
− Hair with bulbs from the center of the lesion, taken with tweezers;
− Swabs of pus taken with a dry, sterile brush.

The improper collection of material may cause false-negative results of mycological examination. There was no fluorescence of disease lesions in the light of a Wood’s lamp. From some of the collected diagnostic material, preparations were performed for direct microscopic examination. The material was treated with 10–20% KOH with the addition of 40% DMSO (dimethyl sulfoxide) and examined in a light or phase-contrast microscope at 200- and 400-fold magnification, looking for mycelial hyphae or spores (ZEISS Axio Lab.A1 Microscope and ZEISS Axiocam 105 color Microscope Camera, Carl Zeiss Microscopy GmbH, Jena, Germany). ZEN Microscopy Software 3.9 was used for microscopic imaging and data archiving (Carl Zeiss Microscopy GmbH, Jena, Germany). Additionally, the collected material was treated with Calcofluor and assessed by fluorescence microscopy (characteristic fluorescence of fungal hyphae). The cultivation of the diagnostic material to identify the fungus species was carried out on Sabouraud Dextrose Agar (SDA) (Aura, Zawroty, Poland) with the addition of chloramphenicol and actidione at the temperatures of 25 ℃ and 37 ℃, for a period of 3–6 weeks. The species identification of dermatophytes grown on media was made based on macroscopic and microscopic features visible in the obtained cultures (characteristic hyphae and spores) (Figure 4 and Figure 5). The results are presented as descriptive statistics (ranges and percentages).

## 3. Results

This study involved 40 people, cattle breeders and their family members, aged from 1 to 75 years. The average age of all patients was 21 years. Children constituted half of the study population. Among them, the largest group consisted of 15 children aged up to 5 years. Men predominated and constituted 60% of the study group (24 patients). In adulthood, dermatophytosis was most often observed between the ages of 31 and 35 (Figure 6).

**Figure 3:**
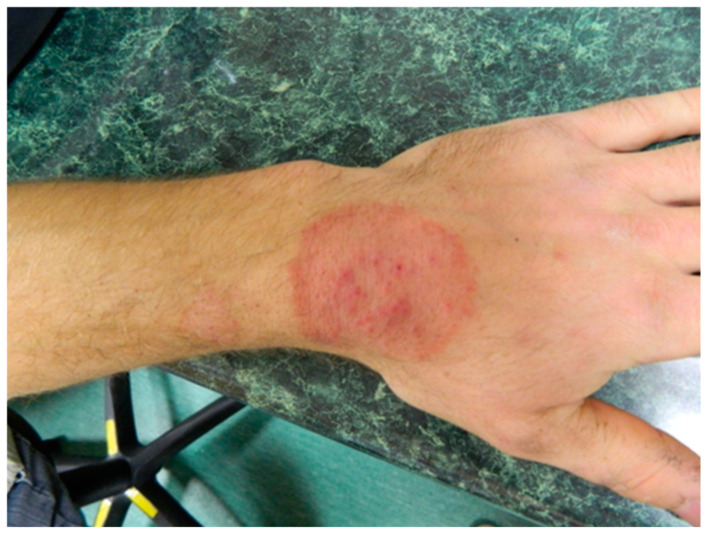
Clinical picture of glabrous skin mycosis caused by *T. verrucosum* infection (courtesy of Honorata Kubisiak-Rzepczyk).

**Figure 4:**
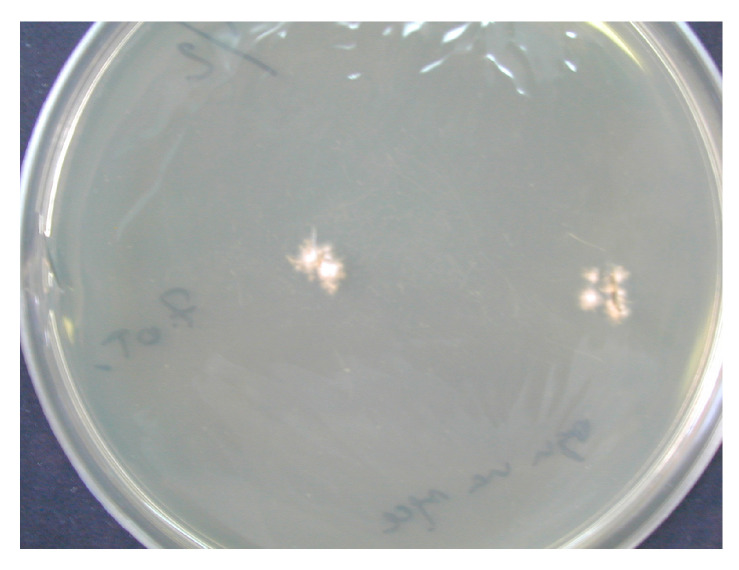
Culture of *T. verrucosum* on SDA (courtesy of Honorata Kubisiak-Rzepczyk).

**Figure 5:**
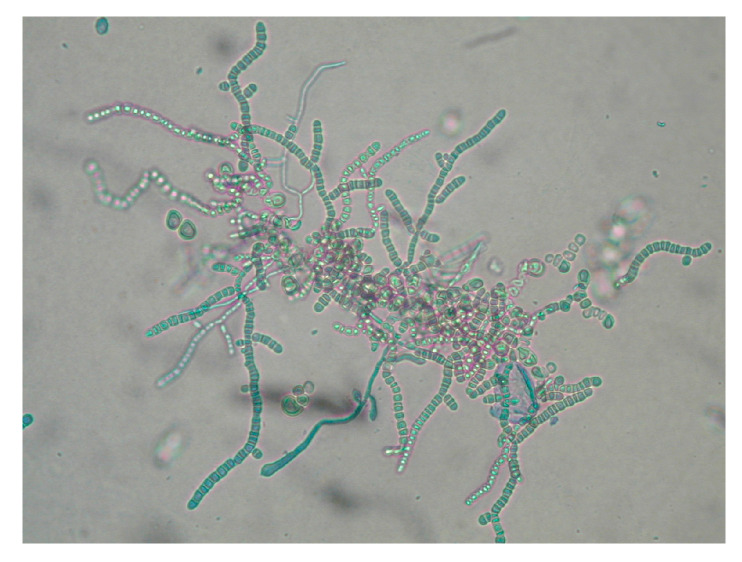
Microscopic examination (magnification ×400) (courtesy of Honorata Kubisiak-Rzepczyk).

**Figure 6:**
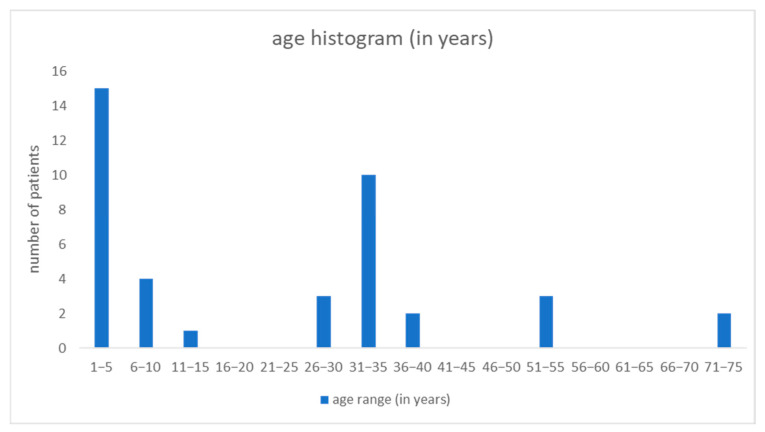
Number of patients in particular age groups.

**Figure 7:**
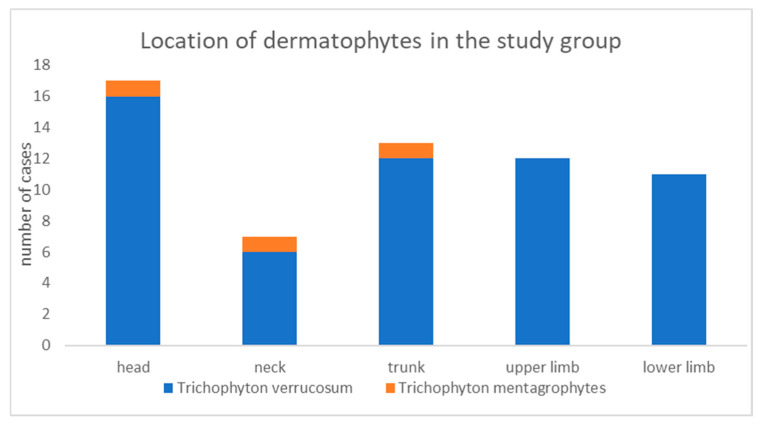
General localization of dermatophytes.

Fungal skin infections in people directly or indirectly involved with cattle breeding were located in various parts of the body. Skin lesions caused by *T. verrucosum* (Figure 3) were most frequently observed on the head, followed by the trunk, the upper limb, the lower limb and the neck. *T. mentagrophytes* infection was diagnosed in one case and was found on the scalp, neck and trunk (Figure 7).

A detailed analysis of skin infections made it possible to determine the frequency of dermatophytes in specific parts of the body. Dermatophytosis was present only in one location in 14 patients. The distribution of skin mycosis in two locations was observed in nine cases, and in three places in the remaining three patients. The most common site of T. verrucosum infection was the stomach, followed by the hairy scalp, back, neck and thigh. Subsequently, less numerous skin lesions caused by this dermatophyte were observed in other parts of the body. T. mentagrophytes diagnosed in one patient was localized on the skin of the cheeks, neck, chest, stomach and back (Table 2).

**Figure 8:**
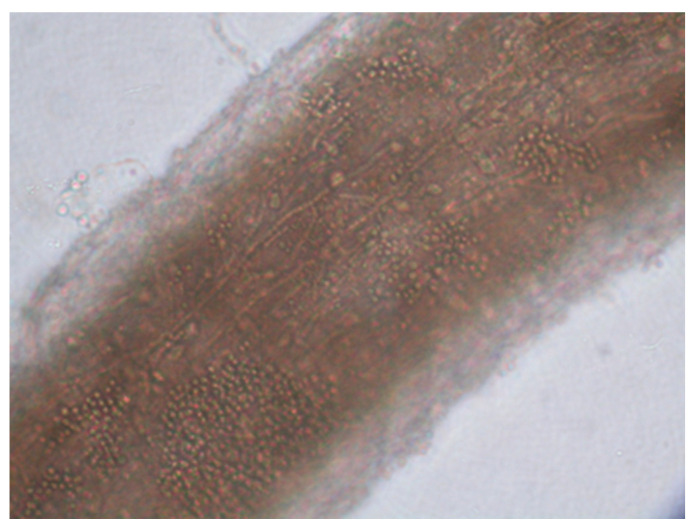
Presence of large spores on the surface of the hair (light microscope, magnification ×400) (courtesy of Honorata Kubisiak-Rzepczyk).

**Figure 9:**
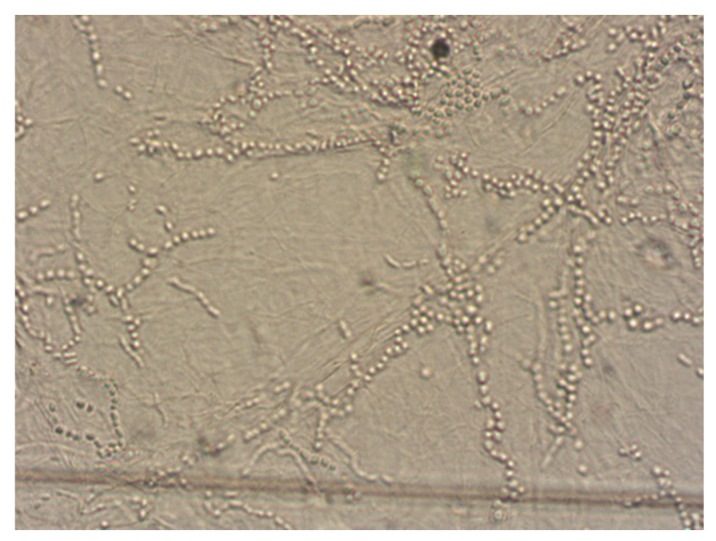
Epidermal scales-hyphae in direct preparation (light microscope, magnification ×400) (courtesy of Honorata Kubisiak-Rzepczyk).

The direct microscopic examination allowed for the identification of structural elements (hyphae and/or spores) in 26 patients (65%) (Figure 8 and Figure 9). A positive *T. verrucosum* result was found in 25 patients, and *T. mentagrophytes* was positive in only one case. The microscopic image of *T. verrucosum* showed microconidia forming chlamydospores in the form of long chains. In the case of *T. mentagrophytes* infection, microconidia were arranged singly along unbranched hyphae. In order to identify the dermatophyte species, the culture was performed regardless of the direct examination result. *T. verrucosum* was confirmed in 39 patients (97.5%), and *T. mentagrophytes* infection was diagnosed in one patient. The highest incidence of infection occurred in late autumn and winter. All patients with *T. mentagrophytes* and *T. verrucosum* infections came from the same geographical area and the same climatic conditions.

## 4. Discussion

The first cases of *T. verrucosum* from cattle were reported in 1959 in the United States in a group of dairy workers from Chicago [38]. Fungal skin infections in humans constitute an important clinical problem in some countries. The prevalence of dermatophytoses is diverse and depends on many factors, such as the socio-economic and sanitary conditions, increasing migration, development of tourism, immunological competence of the host and pathogenicity of the infectious agent, as well as the availability of medical resources and treatment [39]. The estimated data indicate that dermatophytosis affects over 20–25% of the world’s population [40]. Ringworm in cattle is highly contagious for breeders, veterinary doctors and veterinary technicians, as well as farm workers and employees transporting animals [41]. The main reservoir of *T. verrucosum* is cattle, and the fungus is rarely transmitted by other animals such as dogs, pigs, sheep, cats, goats and horses [42]. Cattle of all ages can be asymptomatic carriers of *T. verrucosum* and *T. mentagrophytes*. However, the occurrence of asymptomatic carriers of both dermatophytes is significantly higher in young animals under the 6th month of age [13]. Infection is facilitated by easy, direct contact with animals and microinjuries to the skin. Dermatophytes penetrate hair follicles and the stratum corneum, digest keratin and produce hyphae and conidia.

Our study showed that half of the patients were under 16 years of age. It is worth noting that in the study population, as many as 37.5% of children were under 6 years old. Failure to comply with the hygiene rules by adults in direct contact with the animals and the immediate environment was probably an indirect route of infection in young children. Some parents carried their children into the barn, where they could become directly infected. Similar cases of dermatophytosis in young children caused by being around cattle or playing in the cattle barn have been described by other authors [43,44]. The older children were often in the vicinity of cattle in the cowshed and outside them. The second largest group of the patients were adults directly associated with cattle breeding. In the study group, the most common sites of *T. verrucosum* infection and the only case of *T. mentagrophytes* infection were various areas of the trunk, followed by the head and upper and lower limbs. In children, the most common location of lesions was the head, and in adults, it was the upper limb. The clinical image of skin lesions caused by both pathogens is similar; however, in the case of *T. mentagrophytes* infection, the inflamed lesions usually affect the glabrous skin of the trunk and face, and the scalp less frequently [45]. In patients with primary and acquired immunodeficiency, dermatophytosis leads to the development of severe forms of disease, including extensive or invasive deep skin changes. The clinical manifestations of the infection may be nonspecific; lymph nodes and other organs may be affected [46,47,48,49].

The diagnostic standard of dermatophytosis is a direct microscopic examination and culture of material taken from fungus-affected skin or hair [50]. Some mycological laboratories for species identification use sensitive molecular methods [22,51]. The results obtained in this study showed that the direct microscopic examination allows for the identification of fungal hyphae and spores only in some patients. The diagnostic usefulness of the method is estimated at 90.5% [23]. Regardless of the light and fluorescence microscopy results, the fungal species were identified from the culture based on the characteristic appearance of hyphae and spores. In all patients, a skin infection with a specific dermatophyte was confirmed. The culture method is widely used for the diagnosis of fungi around the world [50]. Molecular methods used in diagnostics have a sensitivity of 76.7% [23]. Mycological diagnosis should be supplemented with a detailed professional and social interview, including holidays, free time and contact with cattle [42]. 

Vaccination programs implemented in many countries have proven effective and have contributed to low rates of infections in farm animals in central and northern Europe. Infections caused by *T. verrucosum* are still relatively more common in southern Europe and Arab countries [52]. Dermatophytosis in cattle is most often self-healing and does not require antifungal therapy. The disease can leave permanent changes on the skin, which reduces its quality and use in industry [7]. The treatment of dermatophytosis in humans should be carried out under the strict supervision of a dermatologist who decides on the choice of a drug and evaluates its effects. A local treatment of mycosis of smooth and hairy skin with the use of antifungal preparations in the form of shampoos and ointments was carried out with all the patients included in the study. During and after the treatment period, all patients underwent regular, scheduled visits to the dermatology clinic. In no case was it necessary to use oral medications. Our experience has shown that compliance with medical recommendations results in the high effectiveness of a local treatment. Advanced and extensive skin lesions require the implementation of systemic therapy [33]. 

The presented article is a retrospective study. It used available information on the confirmed dermatophytosis from cattle. Taking into account the cattle population in Greater Poland, the number of illnesses among cattle breeders and their family members over the course of over six and a half years does not seem to be high. However, it may pose a significant health, social and economic problem for people affected by this disease. 

So far, there have been few published works on *T. verrucosum* infections in cattle breeders in Poland, including those concerning the genomic variability of strains. This publication is the first study from Greater Poland concerning cattle-to-human superficial mycoses in this group of people.

## 5. Conclusions

Dermatophytosis in humans can be caused by direct contact with dermatophyte-infected cattle and elements of the animal environment, as well as indirectly due to non-compliance with hygiene rules. Skin changes in humans associated with cattle are more often diagnosed in small children and men directly engaged in cattle breeding. The dominant etiological factor of the superficial fungal skin infection associated with cattle is *T. verrucosum*, which mainly affects the scalp in children and upper limbs in adult men.

The preventive strategies to effectively control fungal infections in humans and cattle should include systematic epidemiological studies, hygiene practices, improved husbandry conditions and early treatment of infected animals and people. The vaccination of young calves has fundamental therapeutic and preventive importance.

## Figures and Tables

**Figure 1:**
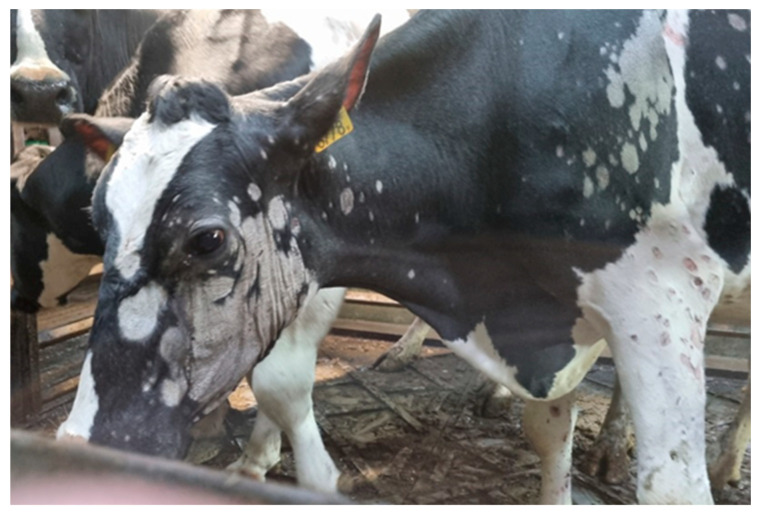
Skin lesions in the course of *T. verrucosum* infection observed in cattle.

**Figure 2:**
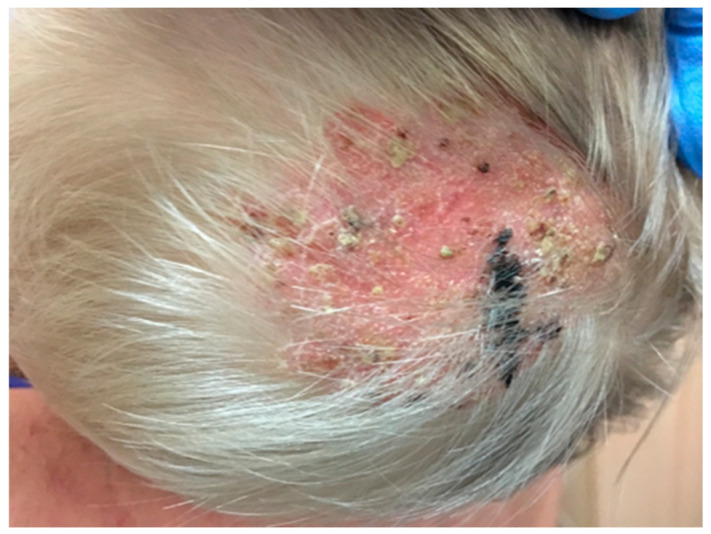
Ringworm of the scalp caused by *T. verrucosum* (courtesy of Honorata Kubisiak-Rzepczyk).

**Table 2:** Detailed distribution of dermatophytes on the skin.

General Location	Detailed Location	Number of Cases [%]		Number of Dermatophyte Species
Head	Hairy scalp	8	[11.11]	*Trichophyton verrucosum*–8
	Eye area	2	[2.78]	*Trichophyton verrucosum*–2
	Cheeks	5	[6.94]	*Trichophyton verrucosum*–4
				*Trichophyton mentagrophytes*–1
	Ear	1	[1.39]	*Trichophyton verrucosum*–1
	Beard	1	[1.39]	*Trichophyton verrucosum*–1
Neck	Neck	7	[9.72]	*Trichophyton verrucosum*–6
				*Trichophyton mentagrophytes*–1
Trunk	Chest	5	[6.94]	*Trichophyton verrucosum*–4
				*Trichophyton mentagrophytes*–1
	Back	8	[11.11]	*Trichophyton verrucosum*–7
				*Trichophyton mentagrophytes*–1
	Stomach	10	[13.88]	*Trichophyton verrucosum*–9
				*Trichophyton mentagrophytes*–1
Upper limb	Palm	2	[2.78]	*Trichophyton verrucosum*–2
	Palms	2	[2.78]	*Trichophyton verrucosum*–2
	Forearm	3	[4.17]	*Trichophyton verrucosum*–3
	Forearms	4	[5.56]	*Trichophyton verrucosum*–4
	Arm	2	[2.78]	*Trichophyton verrucosum*–2
	Arms	1	[1.39]	*Trichophyton verrucosum*–1
Lower limb	Shank	1	[1.39]	*Trichophyton verrucosum*–1
	Shanks	1	[1.39]	*Trichophyton verrucosum*–1
	Thigh	5	[6.94]	*Trichophyton verrucosum*–5
	Thighs	3	[4.17]	*Trichophyton verrucosum*–3
	Buttocks	1	[1.39]	*Trichophyton verrucosum*–1

## Data Availability

Data are contained within the article.

## References

[1] Statista. https://www.statista.com/statistics/263979/global-cattle-population-since-1990.

[2] List of Letter Codes for Cattle. https://www.bing.com/search?q=Wykaz+kod%C3%B3w+literowych+do+oznaczania+ras+byd%C5%82a&qs=n&form=QBRE&sp=-1&lq=0&pq=wykaz+kod%C3%B3w+literowych+do+oznaczania+ras+byd%C5%82a&sc=0-46&sk=&cvid=80A494889A3646FA8E6D8137EB2C78D4&ghsh=0&ghacc=0&ghpl=breeds.

[3] Statista. https://www.statista.com/statistics/1405768/poland-number-of-cattle-and-cows.

[4] Statistics Poland. https://stat.gov.pl/files/gfx/portalinformacyjny/pl/defaultaktualnosci/5508/5/25/1/poglowie_bydla_wedlug_stanu_w_grudniu_2023_r..pdf.

[5] Smith M.B., McGinnis M.R. (2011). Chapter 82—Dermatophytosis. Tropical Infectious Diseases: Principles, Pathogens and Practice.

[6] Papini R., Nardoni S., Fanelli A., Mancianti F. (2009). High infection rate of *Trichophyton verrucosum* in calves from Central Italy. Zoonoses Public Health.

[7] Hameed K., Riaz C.F., Nawaz M.A., Naqvi S.M.S., Gräser Y., Kupsch C., Pasquetti M., Rossi L., Molinar Min A.R., Tizzani P. (2017). *Trichophyton verrucosum* infection in livestock in the Chitral district of Pakistan. J. Infect. Dev. Ctries..

[8] Maurice M.N., Kazeem H.M., Kwanashie C.N., Maurice N.A., Ngbede E.O., Adamu H.N., Mshelia W.P., Edeh R.E. (2016). Equine Dermatophytosis: A Survey of Its Occurrence and Species Distribution among Horses in Kaduna State, Nigeria. Scientifica.

[9] Łagowski D., Gnat S., Nowakiewicz A., Osińska M., Trościańczyk A., Zięba P. (2019). In search of the source of dermatophytosis: Epidemiological analysis of *Trichophyton verrucosum* infection in llamas and the breeder (case report). Zoonoses Public Health.

[10] Kuttin E.S., Alhanaty E., Feldman M., Chaimovits M., Müller J. (1986). Dermatophytosis of camels. J. Med. Vet. Mycol..

[11] Robi D.T., Mossie T., Temteme S. (2023). Eukaryotic Infections in Dairy Calves: Impacts, Diagnosis, and Strategies for Prevention and Control. Vet. Med. Res. Rep..

[12] Radostits O.M., Gay C.C., Hinchcliff K.W., Constable P.D. (2007). Veterinary Medicine: A Textbook of the Diseases of Cattle, Horses, Sheep, Pigs and Goats.

[13] Agnetti F., Righi C., Scoccia E., Felici A., Crotti S., Moretta I., Moretti A., Maresca C., Troiani L., Papini M. (2014). *Trichophyton verrucosum* infection in cattle farms of Umbria (Central Italy] and transmission to humans. Mycoses.

[14] Woodfolk J.A. (2005). Allergy and dermatophytes. Clin. Microbiol. Rev..

[15] Guo Y., Ge S., Luo H., Rehman A., Li Y., He S. (2020). Occurrence of *Trichophyton verrucosum* in cattle in the Ningxia Hui autonomous region, China. BMC Vet. Res..

[16] Courtellemont L., Chevrier S., Degeilh B., Belaz S., Gangneux J.P., Robert–Gangneux F. (2017). Epidemiology of *Trichophyton verrucosum* infection in Rennes University Hospital, France: A 12–year retrospective study. Med. Mycol..

[17] Lund A., Bratberg A.M., Næss B., Gudding R. (2014). Control of bovine ringworm by vaccination in Norway. Vet. Immunol. Immunopathol..

[18] Singh I., Kushwaha R.K. (2010). Dermatophytes and related keratinophilic fungi in soil of parks and agricultural fields of uttar pradesh, India. Indian J. Dermatol..

[19] Zienicke H., Korting H.C. (1989). Intrafamilial transmission of *Trichophyton verrucosum* to a newborn. Mycoses..

[20] Roman C., Massai L., Gianni C., Crosti C. (2001). Case reports. Six cases of infection due to *Trichophyton verrucosum*. Mycoses.

[21] Swai E.S., Sanka P.N. (2012). Bovine Dermatophytosis Caused by *Trychophyton verrucosum*: A Case Report. Vet. World.

[22] Gnat S., Łagowski D., Nowakiewicz A., Dyląg M., Osińska M., Sawicki M. (2021). Detection and identification of dermatophytes based on currently available methods—A comparative study. J. Appl. Microbiol..

[23] Aboul–Ella H., Hamed R., Abo–Elyazeed H. (2020). Recent trends in rapid diagnostic techniques for dermatophytosis. Int. J. Vet. Sci. Med..

[24] Bednarski M. (2015). Choroby Bydła Podstawy Diagnostyki i Terapii.

[25] Gnat S., Łagowski D., Nowakiewicz A., Trościańczyk A., Zięba P. (2018). Infection of *Trichophyton verrucosum* in cattle breeders, Poland: A 40–year retrospective study on the genomic variability of strains. Mycoses.

[26] Araújo C.R., Miranda K.C., Fernandes Ode F., Soares A.J., Silva Mdo R. (2009). In Vitro susceptibility testing of dermatophytes isolated in Goiania, Brazil, against five antifungal agents by broth microdilution method. Rev. Inst. Med. Trop. São Paulo.

[27] Aly M.M., Bafiel S. Screening for Antimicrobial Activity of Some Medicinal Plants in Saudi Arabia. Proceedings of the World Conference on Medical and Aromatic.

[28] Gołyński M., Lutnicki K., Kostro K. (2012). Effect of Oral Administration of Zinc Sulphate with Simultaneous Use of Nonspecific Immunostimulation on the Course of Trichophytosis in Beef Cattle. J. Vet. Res..

[29] Nisbet C., Yarim G.F., Ciftci G., Arslan H.H., Ciftci A. (2006). Effects of trichophytosis on serum zinc levels in calves. Biol. Trace Elem. Res..

[30] Pasa S., Kıral F., Kodu Y. (2009). Serum zinc and vitamin A concentrations in calves with dermatophytosis. Kafkas Univ. Vet. Fak. Derg..

[31] Değirmençay S., Eroğlu M.S., Kirbas A., Adiguzel M.A. (2023). 25–Hydroxyvitamin D and Parathyroid Hormone Concentrations in Cattle with Dermatophytosis. Firat Univ. J. Health Sci..

[32] Hryncewicz–Gwóźdź A. (2023). Dermatofitozy. Medycyna Praktyczna dla Lekarzy. https://www.mp.pl/interna/chapter/B16.II.18.142.1.1.

[33] Nowicki R.J., Romaszkiewicz A., Rudnicka L., Olszewska M., Sar–Pomian M., Rakowska A. (2022). Infekcje dermatofitowe. Współczesna Dermatologia.

[34] Pal M. (2017). Dermatophytosis in an adult cattle due to *Trichophyton verrucosum*. Anim. Husb. Dairy Vet. Sci..

[35] Mikaili A., Chalabi M., Ghashghaie A., Mostafaie A. (2012). Immunization against bovine dermatophytosis with live *Trichophyton verrucosum*. Afr. J. Microbiol. Res..

[36] Lund A., Deboer D.J. (2008). Immunoprophylaxis of dermatophytosis in animals. Mycopathologia.

[37] Abo–Elyazeed H., Soliman R., Hassan H., El–Seedy F.R., Aboul–Ella H. (2023). Development, preparation, and evaluation of a novel non–adjuvanted polyvalent dermatophytes vaccine. Sci. Rep..

[38] Ortiz D.A., Ren P. (2019). Answer to July 2019 Photo Quiz. J. Clin. Microbiol..

[39] Lee Y.W., Yun S.J., Lee J.B., Kim S.J., Lee S.C., Won Y.H. (2013). Clinical and mycological studies on dermatomycosis (2001–2010). Korean J. Mycol..

[40] Havlickova B., Czaika V.A., Friedrich M. (2008). Epidemiological trends in skin mycoses worldwide. Mycoses.

[41] Wagdy R.E., Mohamed E.A. (2016). Identification of different Dermatophytes isolated from cattle, cats and horses suffered from skin lesions. Alex. J. Vet. Sci..

[42] Schumny U., Wiegand C., Hipler U.C., Darr–Foit S., Peckruhn M., Uhrlaß S., Nenoff P., Elsner P. (2020). Occupational *Trichophyton verrucosum* infection in a cattle farmer. Hautarzt.

[43] Jiang Y., Zhan P., Al–Hatmi A.M.S., Shi G., Wei Y., van den Ende A.H.G.G., Meis J.F., Lu H., de Hoog G.S. (2019). Extensive tinea capitis and corporis in a child caused by *Trichophyton verrucosum*. J. Med. Mycol..

[44] Maslen M.M. (2000). Human cases of cattle ringworm due to *Trichophyton verrucosum* in Victoria, Australia. Australas. J. Dermatol..

[45] Švarcová M., Větrovský T., Kolařík M., Hubka V. (2023). Defining the relationship between phylogeny, clinical manifestation, and phenotype for *Trichophyton mentagrophytes*/*interdigitale* complex; a literature review and taxonomic recommendations. Med. Mycol..

[46] Rouzaud C., Hay R., Chosidow O., Dupin N., Puel A., Lortholary O., Lanternier F. (2015). Severe Dermatophytosis and Acquired or Innate Immunodeficiency: A Review. J. Fungi.

[47] Lanternier F., Cypowyj S., Picard C., Bustamante J., Lortholary O., Casanova J.L., Puel A. (2013). Primary immunodeficiencies underlying fungal infections. Curr. Opin. Pediatr..

[48] Gega A., Ketsela G., Glavin F.L., Soldevilla–Pico C., Schain D. (2010). Majocchi’s granuloma after antithymocyte globulin therapy in a liver transplant patient. Transpl. Infect. Dis..

[49] Muñoz–Pèrez M.A., Rodriguez–Pichardo A., Camacho F., Rios J.J. (2000). Extensive and deep dermatophytosis caused by *Trichophyton mentagrophytes* var. interdigitalis in an HIV–1 positive patient. J. Eur. Acad. Dermatol. Venereol..

[50] Weitzman I., Summerbell R.C. (1995). The dermatophytes. Clin. Microbiol. Rev..

[51] Spanamberg A., Ravazzolo A.P., Araujo R., Franceschi N., Ferreiro L. (2023). Bovine ringworm—Detection of *Trichophyton verrucosum* by SYBR–Green real–time PCR. Med. Mycol. Case Rep..

[52] O’Gorman S.M., Britton D., Collins P. (2015). An uncommon dermatophyte infection: Two cases of cutaneous infection with *Trichophyton verrucosum*. Clin. Exp. Dermatol..

